# Incidence and prevalence of kidney replacement therapy in Central and Eastern Europe—trends from the ERA Registry

**DOI:** 10.1093/ndt/gfaf268

**Published:** 2026-01-07

**Authors:** Marjolein Bonthuis, Anneke Kramer, Sevcan A Bakkaloğlu, Jaakko Helve, Nikola Gjorgjievski, Halima Resic, Anders Åsberg, Nicos Mitsides, Alicja M Dębska-Ślizień, Kirill S Komissarov, Viktorija Kuzema, Nurhan Seyahi, Belén Ponte, Edita Ziginskiene, Mirjana Lausevic, Ivan Rychlík, Mai Ots-Rosenberg, Evgueniy Vazelov, George Moustakas, Adrián Okša, Ariana Strakosha, Liliana Garneata, Dajana Katicic, Roser Torra, Alberto Ortiz, Vianda S Stel

**Affiliations:** ERA Registry, Amsterdam UMC location University of Amsterdam, Medical Informatics, Amsterdam, The Netherlands; Amsterdam Public Health Research Institute, Quality of Care, Amsterdam, The Netherlands; ERA Registry, Amsterdam UMC location University of Amsterdam, Medical Informatics, Amsterdam, The Netherlands; Amsterdam Public Health Research Institute, Quality of Care, Amsterdam, The Netherlands; Gazi University, Faculty of Medicine, Department of Pediatric Nephrology, Ankara, Türkiye; Finnish Registry for Kidney Diseases, Finnish Institute for Health and Welfare, Helsinki, Finland; Abdominal Center Nephrology, University of Helsinki and Helsinki University Hospital, Helsinki, Finland; University Clinic of Nephrology, Skopje, North Macedonia; Faculty of Medicine, University ‘SS Cyril and Methodius’ Skopje, Skopje, North Macedonia; Society of Nephrology and Dialysis of Bosnia and Herzegovina, Sarajevo, Bosnia and Herzegovina; Department of Transplantation Medicine, Oslo University Hospital - Rikshospitalet, Oslo, Norway; Cyprus Renal Registry, Health Monitoring Unit, Ministry of Health, Nicosia, Cyprus; University of Cyprus, Shacolas Education Centre for Clinical Medicine, Nicosia, Cyprus; Nephrology Department, Nicosia General Hospital, State Healthcare Services Organisation, Nicosia, Cyprus; Medical University of Gdańsk, Department of Nephrology, Transplantology and Internal Diseases, Gdańsk, Poland; State Institution “Minsk Scientific and Practical Center for Surgery, Transplantology and Hematology”, Minsk, Belarus; Department of Nephrology, Pauls Stradins Clinical University Hospital, Riga, Latvia; Department of Internal Diseases, Riga Stradins University, Riga, Latvia; Istanbul Üniversity-Cerrahpasa, Cerrahpasa Medical Faculty, Istanbul, Türkiye; Department of Medicine, Division of Nephology and Hypertension, Geneva University Hospitals, Geneva, Switzerland; Nephrology Department, Medical Academy, Lithuanian University of Health Sciences, Kaunas, Lithuania; Lithuanian Nephrology, Dialysis and Transplantation Association, Lithuania; Medical Faculty, University of Belgrade, Nephrology Clinic, University clinical center of Serbia, Belgrade, Serbia; Department Internal Medicine, Third Faculty of Medicine, Charles University and University Hospital Kralovske Vinohrady, Prague, Czechia; Department of Internal Medicine, Tartu University Hospital, Tartu, Estonia; Department of Internal Diseases, University “Prof. Dr Asen Zlatarov”, Burgas, Bulgaria; Nephrology Department, General Hospital of Athens “G. Gennimatas”, Athens, Greece; Slovak Medical University, Faculty of Medicine, Bratislava, Slovakia; Service of Nephrology, UHC “Mother Teresa”, Tirana, Albania; Carol Davila University of Medicine and Pharmacy, Bucharest, Romania; Department of Internal Medicine and Nephrology, R Carol Davila Teaching Hospital of Nephrology, Bucharest, Romania; Croatian Society of Nephrology, Dialysis and Transplantation, Croatia; Inherited Kidney Diseases, Nephrology Department, Fundació Puigvert, Institut de Recerca Sant Pau, Universitat Autònoma de Barcelona, Barcelona, Spain; Department of Nephrology and Hypertension, IIS-Fundacion Jimenez Diaz UAM, Madrid, Spain; RICORS2040, Madrid, Spain; ERA Registry, Amsterdam UMC location University of Amsterdam, Medical Informatics, Amsterdam, The Netherlands; Amsterdam Public Health Research Institute, Quality of Care, Amsterdam, The Netherlands

**Keywords:** Central and Eastern Europe, dialysis, incidence, kidney transplantation, prevalence

## Abstract

**Background and hypothesis:**

Kidney replacement therapy (KRT) practices in Europe are heterogeneous, with apparent differences between Western and Central/Eastern Europe. However, time trends in KRT incidence and prevalence in Central and Eastern Europe have not been previously reported. Therefore, we aimed to describe trends in incidence and prevalence of KRT in Central and Eastern Europe from 2010 to 2021.

**Methods:**

Data on incident and prevalent KRT patients from 19 Central and Eastern European countries between the years 2010 and 2021 were derived from the European Renal Association (ERA) Registry. Time trends were calculated using JoinPoint regression.

**Results:**

Overall, KRT incidence increased at 1.5% [95% confidence interval (CI): +0.7 to +2.6%] per year from 106.3 per million population (pmp) in 2010 to 119.6 pmp in 2019. However, trends differed within the region. While in Bosnia and Herzegovina KRT incidence significantly decreased from 2010 to 2019, it remained stable in nine and increased in eight countries. The overall KRT prevalence increased at 5.1% (95% CI: +4.5 to +5.7%) per year from 426.2 pmp in 2010 to 651.2 pmp in 2019. KRT prevalence increased in all countries, except for Belarus where it remained stable, and was mainly attributable to increases in the prevalence of kidney transplantation. The COVID-19 pandemic did not have a major impact on KRT incidence and prevalence in the region, as most trends remained until 2021.

**Conclusions:**

Although we found an overall increase in KRT incidence and prevalence in the region, large country variations remain, much larger than observed in Western Europe. The results of this study can help to define country-specific priorities for the optimization of KRT care in Central and Eastern Europe.

## Graphical Abstract

**Graphical Abstract:**
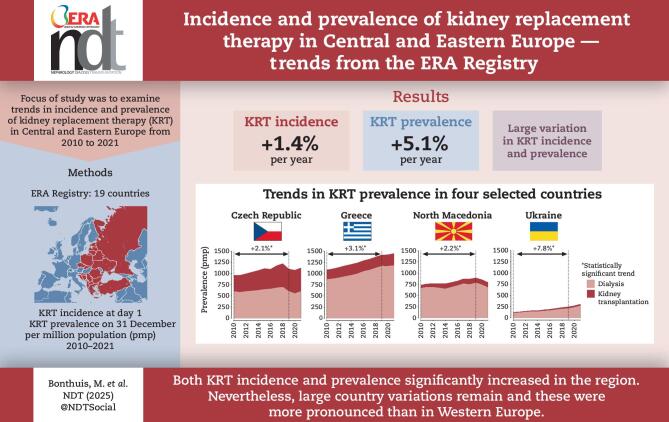

KEY LEARNING POINTS **What was known:**

- The burden of kidney failure and the use of kidney replacement therapy (KRT) vary widely across countries and regions in Europe. Economic factors are considered to be the driving force behind differences in access to KRT, with lower access in lower-income countries in Central and Eastern Europe.
- Owing to population aging and the increasing prevalence of diabetes mellitus and hypertension, the number of patients with kidney failure requiring KRT is increasing.
- Previous ERA Registry studies have shown that after a period of stabilization, KRT incidence increased from 2011 to 2017, whereas KRT prevalence continuously increased in the same period. However, these studies mainly included Western European countries only, and such trends are unknown for Central and Eastern Europe.

**This study adds:**

- Our study provides the first overview of KRT incidence and prevalence trends over a decade for Central and Eastern Europe using data from the ERA Registry.
- In Central and Eastern Europe, both KRT incidence (+1.5% per year) and prevalence (+5% per year) increased significantly from 2010 to 2019, but were lower than in Western Europe. However, there was large country variation in KRT incidence and prevalence across the region, much larger than the variation observed in Western Europe.
- Although hemodialysis was the dominant treatment modality in most countries, increases in KRT prevalence were mainly attributable to increases in the prevalence of kidney transplantation. Nevertheless kidney transplantation numbers are still considerably lower than in Western Europe.

**Potential impact:**

- This study is the first to provide a complete overview on trends in KRT incidence and prevalence in Central and Eastern Europe. This may assist policy makers and the medical community in healthcare planning, and to enhance public awareness for kidney disease and treatment in the region.
- The large country variations in KRT practices might help to define country-specific priorities for the optimization of kidney care, including further development of kidney transplantation programs that should be given high priority.

## INTRODUCTION

Despite huge efforts to accomplish equitable access to high-quality healthcare in Europe [1], large disparities in the utilization of kidney replacement therapy (KRT) for patients with kidney failure remain [2, 3], mainly between Western and Eastern Europe [4, 5]. Several factors, such as the prevalence of chronic kidney disease (CKD) and the presence of CKD risk factors in the general population, but also healthcare infrastructure and public health policies, differences in public awareness and cultural factors might contribute to country differences in the treatment of CKD and the provision of KRT [4, 6, 7]. Nevertheless, economic factors are considered to be the driving force behind differences in access to KRT, with lower access in low-income countries [4, 8, 9].

Previous studies from the European Renal Association (ERA) Registry have shown that in Europe, after years of continuous growth, KRT incidence stabilized between 2000 and 2011, while the incidence slightly increased further from 2011 to 2017, mainly among men aged 65 years or older [10–13]. At the same time, KRT prevalence continuously increased [10, 12]. However, these numbers originated from countries contributing with individual patient data to the ERA Registry, which mainly comprise Western European countries. Most Central and Eastern European countries also participate in the ERA Registry, but the data collection is predominantly conducted on an aggregated country level. Furthermore, trend studies require data collection over a longer follow-up period, which has only recently become available for many Central and Eastern European countries. For the first time, it is now possible to provide an overview of trends in KRT incidence and prevalence over the past decade in this region.

Therefore, we aimed to examine time trends in the incidence and prevalence of KRT in Central and Eastern Europe from 2010

to 2021 using data from the ERA Registry. To this end, we intend to inform all stakeholders about country-specific requirements in the delivery of kidney care to KRT patients in this region.

## MATERIALS AND METHODS

### Data source and patient population

We included incidence and prevalence data on KRT patients between the years 2010 and 2021 from 19 Central and Eastern European countries participating in the ERA Registry. The ERA Registry collects data on KRT patients from population-based national and regional KRT registries across Europe in two ways. First, for several countries data is collected for individual patients. This way, a patient can be followed throughout their KRT trajectory. Furthermore, for other countries, data are collected on an aggregated level and KRT incidence and prevalence numbers are summarized on a country level [14].

Following the definition by Sever *et al.* [4], we included the following Central and Eastern European countries in our study: Bosnia and Herzegovina, Estonia, Greece, Romania and Serbia (using individual patient data), and Albania, Belarus, Bulgaria, Croatia, Cyprus, Czech Republic, Latvia, Lithuania, North Macedonia, Poland, Russia, Slovakia, Türkiye, and Ukraine (using aggregated country level data). Data for Hungary, Moldova, Montenegro, and Slovenia were unavailable or incomplete, and these countries could therefore not be included. Periods of data contribution differed for the included countries (Table 1).

**Table 1:** Unadjusted incidence of KRT at day 1 pmp by country and year.

Country/year	2010	2011	2012	2013	2014	2015	2016	2017	2018	2019	2020	2021	AAPC 2010–2019	AAPC 2010–2021
Albania		76.5	66.5	74.0	88.0	88.0	88.1	89.6	140.1	125.6	130.6	137.6	**+8.0 (+3.9; +12.2)**	**+7.7 (+5.1; +10.3)**
Belarus^a^						80.3		100.3	82.5	93.3	74.2	76.4	+2.6 (−13.3; +21.5)	−2.0 (−8.6; +5.1)
Bosnia and Herzegovina	145.4	120.6	125.4	116.0	120.0	114.4	112.4	107.9	124.6	110.7	115.5	121.2	**−1.9 (−3.5; −0.2)**	−1.1 (−2.4; +0.2)
Bulgaria^b^				165.8	165.9	152.8	156.2	169.5	155.9				−0.6 (−3.6; +2.4)	−0.6 (−3.6; +2.4)
Croatia^b^	140.2	141.7	158.1	156.7	157.3	157.6	179.8	191.2	151.2	147.7	109.3	132.2	+0.3 (−0.3; +2.2)	−1.7 (−4.9; +1.6)
Cyprus				187.1	204.3	191.8	192.5	236.1	255.7	283.8	272.5	283.0	**+7.0 (+3.0; +11.2)**	**+6.2 (+3.9; +8.5)**
Czech Republic^b^	212.0	213.0	227.0	198.5	197.3	232.5	243.2	231.9	226.9	216.3	212.6	243.1	+0.9 (−1.5; +3.3)	+0.8 (−0.9; +2.6)
Estonia	74.6	64.9	80.9	63.7	87.5	86.7	85.1	66.0	72.6	73.9	57.2	76.6	+0.3 (−2.9; +3.6)	−0.6 (−3.1; +2.0)
Greece	190.5	203.0	209.7	215.8	217.8	226.9	250.7	252.2	264.0	268.9	256.9	279.3	**+3.9 (+3.4; +4.5)**	**+3.4 (+2.8; +4.0)**
Latvia	120.7	99.1	88.9	80.6	95.6	96.5	109.6	114.4	107.5	93.3	63.3	93.9	−1.0 (−2.9; +2.7)	−1.5 (−5.8; +2.8)
Lithuania^b^				111.7	104.0	105.4	107.3	119.7	112.5	112.7	86.6	97.5	+1.1 (−1.1; +3.4)	−1.5 (−4.2; +1.3)
North Macedonia	123.1	134.0			132.5	151.8	164.2	180.5	167.7	183.0	184.2	178.4	**+4.4 (+3.1; +5.8)**	**+3.9 (+2.9; +4.9)**
Poland^b^	142.8	133.1	134.6	133.1	119.5	174.3	176.8	196.5	157.4	152.8	124.7	146.0	+3.0 (−0.5; +6.6)	+1.0 (−1.8; +4.0)
Romania^c^	124.4	126.7	150.6	144.7	152.1	159.3	177.1	187.3	182.9	190.7	163.4	173.6	**+5.1 (+3.9; +6.2)**	**+3.0 (+0.9; +5.1)**
Russia^b^	39.5	42.9	48.1	50.1		51.1	59.2	66.6	82.9	87.6	84.5		**+8.9 (+6.9; +11.1)**	**+8.5 (+6.8; +10.2)**
Serbia								91.9	86.9	87.4	77.9	81.3		**−3.5 (−6.5; −0.4)**
Slovakia^b^	167.3	148.8	166.1	157.9	153.3	168.8	153.6	170.5	165.0	121.9	124.9	186.0	−1.2 (−3.6; +1.3)	−0.7 (−3.1; +1.6)
Türkiye			138.6	138.3	147.3	147.3	139.9	146.5	149.2	150.5	138.7	149.5	**+1.1 (+0.1; +2.0)**	+0.5 (−0.3; +1.4)
Ukraine^a,d^	23.0	24.2	27.6	29.8	23.3	23.9	29.3	36.6	37.0	39.9	40.0	53.0	**+5.8 (+2.5; +9.3)**	**+6.8 (+4.3; +9.4)**
Total Eastern Europe	106.3	104.7	93.1	94.2	95.9	102.7	104.8	113.0	120.1	119.6	110.0	121.8	**+1.5 (+0.7; +2.6)**	**+1.1 (+0.3; +2.6)**
Total Western Europe^e^	130.8	130.1	132.1	134.1	137.2	139.1	141.7	143.8	143.8	145.9	134.8	142.3	**+1.4 (+1.1; +1.7)**	**+0.7 (+0.1; +1.2)**

If cells are left empty, data are unavailable.

^a^Patients younger than 18 years of age are not reported for Belarus (2019–2021) and Ukraine (2016–2021).

^b^Data include dialysis patients only for Bulgaria (2013–2015), Croatia (2020–2021), Czech Republic (2012–2021), Lithuania (2013), Poland (2016–2017), Russia (2015–2020), and Slovakia (2010–2021).

^c^The incidence of pre-emptive kidney transplantation is underestimated by ∼30% for the entire period.

^d^Data for 2010 do not include the regions Zakarpattya, Zaporizzha, and Kiev city, and data for 2011 do not include Kiev city.

^e^Including Austria, Belgium (Dutch-speaking and French-speaking), Denmark, Finland, France, Iceland, Norway, Portugal, Spain, Sweden, Switzerland, the Netherlands, UK (England, Northern Ireland, Scotland, and Wales).

Bold AAPC values depict the statistically significant trends.

### Incidence and prevalence

The incidence of KRT was defined as the number of patients starting KRT (i.e. dialysis or pre-emptive kidney transplantation) during a year, whereas the prevalence was defined as the number of patients alive and receiving KRT on 31 December of a particular year. Incidence and prevalence [per million population (pmp)] were calculated by dividing the number of incident or prevalent KRT patients by the general population within the same country and year, multiplied by 1 million. Most registries had full coverage of their general population (Supplementary Table 1). In the case of lower coverage, this was corrected for in the general population statistics. General population data were reported by the collaborating registries or extracted from Eurostat [15].

If provided to the ERA Registry, data on incidence and prevalence were stratified by age group, sex, and primary renal disease (PRD). PRDs were categorized according to the ERA Registry PRD grouping as follows: glomerulonephritis/sclerosis, hypertension (HT), diabetes mellitus (DM), unknown/missing PRD, and other/miscellaneous PRD [14].

### Time trends

We evaluated time trends in the incidence and prevalence using the JoinPoint regression program provided by the Surveillance Research Program of the US National Cancer Institute [16]. In short, this program calculated the slope of the trend with the observed rate as the outcome variable and calendar year as the explanatory variable. To estimate trends, the program requires a minimum of four data points. Trends are expressed as the annual percentage change (APC). For comparison reasons, we only report the average annual percentage change (AAPC), which is a summary measure for the trend over the entire study period [16].

For reasons of comparison we also calculated trends for the overall KRT incidence and prevalence in Western Europe based on data presented in ERA Registry Annual reports [17]. For our main analyses we calculated time trends starting from 2010 until the year 2019 (before the onset of the COVID-19 pandemic). We extended the follow-up period to the year 2021 in sensitivity analyses.

## RESULTS

### Trends in KRT incidence

#### Overall

**Figure 1:**
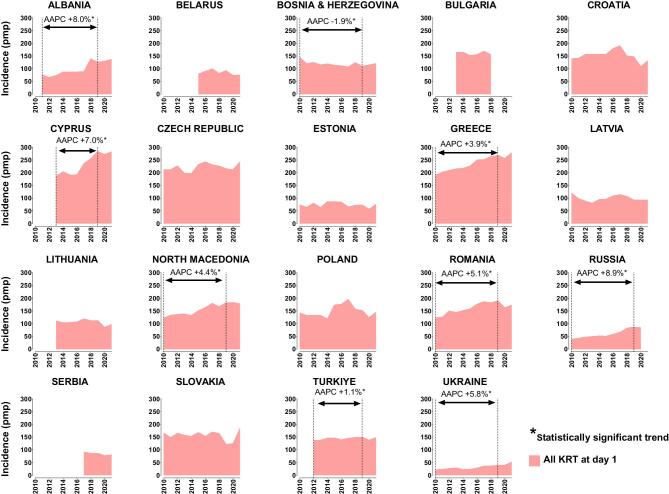
Unadjusted incidence of KRT at day 1 pmp by country and year. Only statistically significant trends in KRT incidence between 2010 and 2019 (AAPCs) are shown in the figure. Patients younger than 18 years of age are not reported for Belarus (2019–2021) and Ukraine (2016–2021). Data include dialysis patients only for Bulgaria (2013–2015), Croatia (2020–2021), Czech Republic (2012–2021), Lithuania (2013), Poland (2016–2017), Russia (2015–2020), and Slovakia (2010–2021). For Romania, the incidence of pre-emptive kidney transplantation is underestimated by ∼30% for 2017–2021. For Ukraine, the data do not include the regions Zakarpattya, Zaporizzha, and Kiev city (2010 and 2011).

Similar to Western Europe, the overall KRT incidence in the region increased on average with 1.5% (95% CI: +0.7 to +2.6%) per year from 106.3 pmp in 2010 to 119.6 pmp in 2019 (Table 1). However, there was large variation in KRT incidence across countries (Fig. 1), ranging from 23.0 pmp in Ukraine to 190.5 pmp in Greece in 2010, and from 39.9 pmp in Ukraine to 283.8 pmp in Cyprus in 2019.

Between 2010 and 2019, KRT incidence significantly increased in eight countries: Albania, Cyprus, Greece, North Macedonia, Romania, Russia, Türkiye, and Ukraine. KRT incidence significantly decreased in Bosnia and Herzegovina (AAPC: −1.9%, 95% CI: −3.5% to −0.6%), whereas it remained stable in the other countries (Fig. 1; Table 1). As the number of pre-emptive kidney transplantations was very low, KRT incidence mainly reflects (hemo)dialysis patients only.

When follow-up was extended to 2021, most of the observed trends in KRT incidence remained. However, for Bosnia and Herzegovina and Türkiye the observed trends were no longer statistically significant, and for Serbia a decreasing trend was observed (Table 1).

#### Age

**Figure 2:**
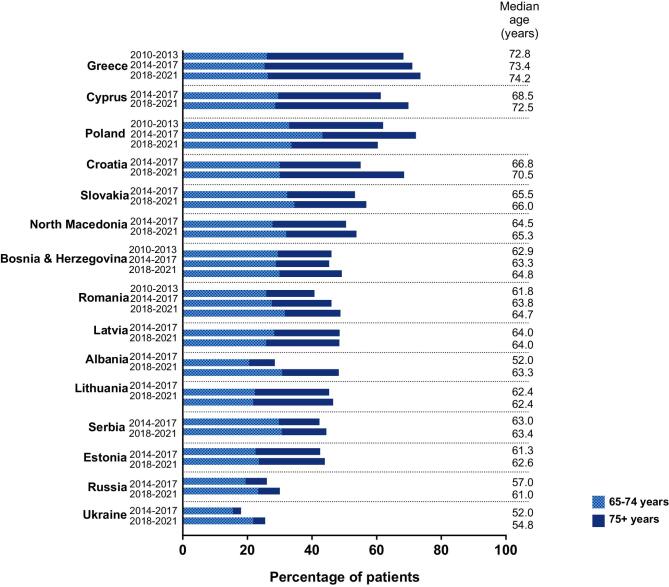
Proportion of patients initiating KRT aged 65 years or older and the median age of incident KRT patients (at day 1) by country and period. Patients younger than 18 years of age are not reported for Ukraine in 2016–2021. Data include dialysis patients only for Croatia (2020–2021), Russia (2015–2020), and Slovakia (2014–2021).

Figure 2 shows the median age of incident KRT patients, as well as the proportion of patients starting KRT at 65 years or older by country and different time periods (2010–2013, 2014–2017, and 2018–2021). Over the time period, median age at KRT initiation varied from 52.0 to 54.8 years in patients from Ukraine to 70.8 to 74.2 years in patients from Greece. Median age tended to increase over time in all countries, except for Latvia and Lithuania (where it remained stable around 62.4–64.0 years). Similarly, we also observed an increase in the proportion of patients initiating KRT at an age of 65 years or older over time. Interestingly, country variation in the age distribution of patients commencing KRT was most pronounced for patients aged 75 years or older (Fig. 2).

#### Sex and primary renal disease

In all countries, more males initiated KRT (Supplementary Fig. 1), without major sex differences in trends over time (Table 2). For some countries, such as Greece and Cyprus, the difference in KRT incidence among males and females was much larger than in Russia and Ukraine where it was almost similar.

**Table 2:** Trends (AAPC) from 2010–2019 in unadjusted KRT incidence (pmp) at day 1 by country stratified by sex and PRD.

	Sex		PRD				
Country	Male	Female	GN	DM	HT	Unknown	Other
Albania	**+7.6 (+3.6; +11.7)**	**+8.6 (+3.0; +14.5)**	+3.4 (−7.3; +15.3)	**+17.8 (+10.9; +25.2)**	**+27.0 (+18.2; +36.5)**	+0.9 (−14.1; +18.6)	+6.7 (−1.5; +15.5)
Belarus^a^	+3.1 (−14.0; +23.6)	+2.0 (−14.1; +21.1)	−0.7 (−17.7; +19.9)	+1.5 (−15.2; +21.6)	+10.4 (−12.4; +39.0)	−13.9 (−72.4; +168.5)	+3.4 (−15.3; +26.2)
Bosnia and Herzegovina	−1.3 (−2.9; +0.3)	**−2.7 (−4.7; −0.6)**	−3.6 (−7.8; +0.9)	−0.1 (−2.1; +2.0)	+1.0 (−2.7; +4.8)	−0.2 (−8.8; +9.2)	−5.3 (−10.8; +0.6)
Bulgaria^b^	+2.0 (−2.8;+ 7.0)	**−3.6 (−6.7; −0.4)**					
Croatia							
Cyprus	**+8.8 (+2.6; +15.4)**	+5.9 (−0.2; +12.5)	**+12.5 (+2.1; +23.8)**	**+11.8 (+2.8; +21.5)**	**+11.7 (+0.6; +24.0)**	−4.3 (−16.4; +9.6)	+1.6 (−5.5; +9.3)
Czech Republic							
Estonia^c^	+1.8 (−2.1; +5.8)	−2.0 (−7.6; +3.9)	+0.9 (−6.0;+8.3)	−0.1 (−3.5; +3.5)	+6.2 (−7.4; +21.9)	N/A	−3.2 (−8.7; +2.5)
Greece	**+4.7 (+4.0; +5.4)**	**+2.7 (+1.8; +3.7)**	+2.0 (−0.3; +4.2)	**+2.7 (+0.4; +5.2)**	+2.6 (0.0; +5.4)	**+6.4 (+4.3; +8.5)**	**+3.5 (+0.8; +6.2)**
Lithuania^b^	+2.4 (−4.9; +10.2)	+0.1 (−7.8; +8.7)	**+8.4 (+1.2; +16.2)**	+5.4 (−3.5; +15.1)	+3.4 (−8.3; +16.5)	−21.4 (−49.0; +21.2)	+7.7 (−9.4; +28.0)
Latvia	+2.0 (−1.1; +5.3)	−2.0 (−6.3; +2.5)	+0.3 (−3.9; +4.7)	+5.0 (−1.1; +11.5)	−1.7 (−13.7; +11.9)	+3.6 (−4.2; +12.0)	−2.0 (−7.6; +3.9)
North Macedonia	**+4.3 (+2.5; +6.2)**	**+4.7 (+1.9; +7.7)**	+1.1 (−3.9; +6.5)	**+7.8 (+4.3; +11.4)**	**+2.1 (+0.2; +4.0)**	+4.5 (−1.1; +10.4)	**+4.9 (+1.5; +8.4)**
Poland			**−5.9 (−7.9; −3.9)**	**−3.0 (−4.9; −0.9)**	**−3.7 (−5.0; −2.3)**	+2.1 (−4.7; +9.2)	+4.0 (−0.1; +8.5)
Romania^d^	**+5.2 (+4.1; +6.4)**	**+4.8 (+3.6; +6.0)**	**−3.9 (−6.0; −0.9)**	**+2.8 (+0.2; +5.4)**	**+8.6 (+4.8; +12.6)**	**+10.0 (+7.1; +12.9)**	**+2.5 (+0.1; +4.9)**
Russia^b^	**+8.3 (+6.2; +10.5)**	**+9.6 (+7.5; +11.7)**	**+6.7 (+2.6; +10.9)**	**+12.5 (+7.6; +17.6)**	**+12.8 (+5.7; +20.3)**	−15.1 (−29.1; +1.6)	**+10.7 (+5.5; +16.1)**
Serbia							
Slovakia^b^	0.0 (−3.1; +3.3)	**−3.0 (−4.7; −1.4)**	−1.5 (−3.8; +0.8)	**−4.1 (−7.9; −0.1)**	−4.8 (−12.2; +3.2)	+2.7 (−1.7; +7.2)	−0.3 (−2.6; +2.1)
Türkiye							
Ukraine^a,e^	**+5.7 (+1.9; +9.7)**	**+6.0 (+1.7; +10.4)**	**+3.9 (+0.4; +7.6)**	**+9.3 (+5.1; +13.7)**	**+11.2 (+5.8; +16.8)**	−1.6 (−31.5; +41.5)	**+6.1 (+2.3; +10.1)**

If cells are left empty, data are unavailable.

^a^Patients younger than 18 years of age are not reported (2019–2021 for Belarus; 2016–2019 for Ukraine).

^b^Data include dialysis patients only (2013–2015 for Bulgaria, 2013 for Lithuania; 2015–2019 for Russia; 2010–2019 for Slovakia).

^c^For 2012 renal vascular disease was not reported separately, but included into hypertension.

^d^The incidence of pre-emptive kidney transplantation is underestimated by ∼30% for the entire period.

^e^Data for 2010 do not include the regions Zakarpattya, Zaporizzha, and Kiev city, and data for 2011 do not include Kiev city.

Bold AAPC values depict the statistically significant trends.

Abbreviation: GN, glomerulonephritis.

**Figure 3:**
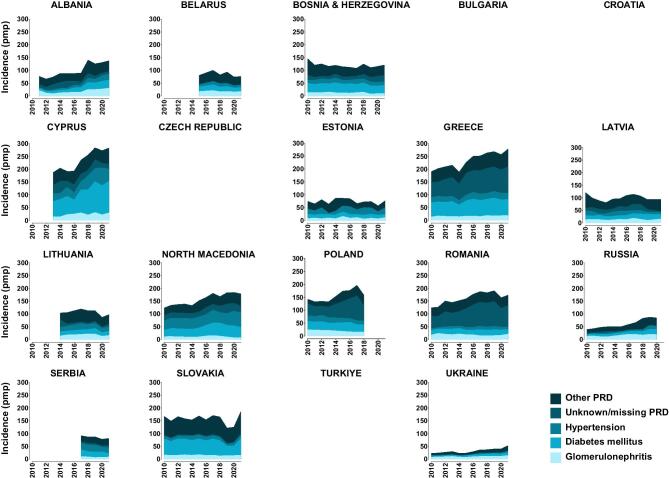
Unadjusted incidence of KRT at day 1 pmp by country and year stratified by PRD. Data are unavailable for Bulgaria, Croatia, Czech Republic, and Türkiye. Patients younger than 18 years of age are not reported for Belarus (2019–2021) and Ukraine (2016–2021). Data include dialysis patients only for Lithuania (2013), Russia (2015–2020), and Slovakia (2010–2021). For Estonia, renal vascular disease was not reported separately, but included into hypertension in 2012. For Romania, the incidence of pre-emptive kidney transplantation is underestimated by ∼30% for 2017–2021. Data for Ukraine do not include the regions Zakarpattya, Zaporizzha, and Kiev city (2010 and 2011).

Although the PRD distribution was highly variable across countries, most patients initiated KRT due to DM or HT (Fig. 3). In countries that showed an overall increasing trend in KRT incidence between 2010 and 2019, the increase was mainly attributed to the increase of patients initiating KRT for kidney failure due to DM or HT, whereas KRT incidence for kidney failure due to glomerulonephritis was more stable over time. For example, in Cyprus each year ∼12% more patients initiated KRT due to DM or HT, resulting in almost half of all patients initiating KRT in 2019 due to DM and 20% due to HT (Table 2).

### Trends in KRT prevalence

#### Overall

**Figure 4:**
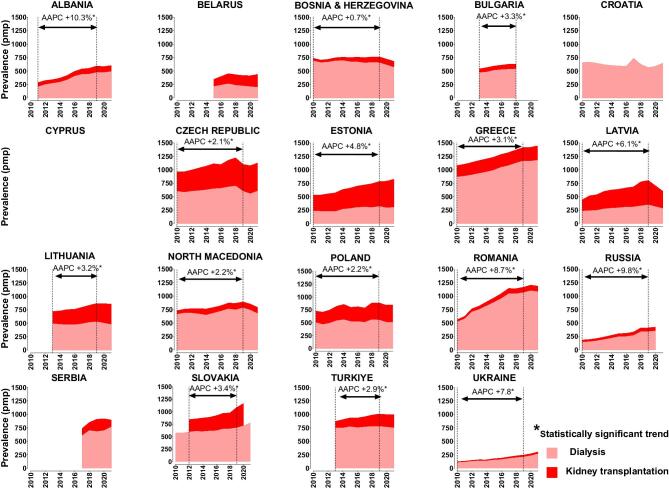
Unadjusted prevalence of KRT (31 December) (pmp) by country and year. Only statistically significant trends in overall KRT prevalence between 2010 and 2019 (AAPCs) are shown in the figure. Data are unavailable for Cyprus. Patients younger than 18 years of age are not reported for Belarus (2019–2021) and Ukraine (2016–2021). For Romania, the overall prevalence of KRT is underestimated by ∼3% due to an estimated 30% underreporting of patients living on a functioning graft. Data for Ukraine do not include the regions Zakarpattya, Zaporizzha, and Kiev city (2010 and 2011). Data for Croatia include dialysis patients only.

The overall KRT prevalence in the region increased on average with 5.1% (95% CI: +4.5% to +5.7%) per year from 426.2 pmp in 2010 to 651.2 pmp in 2019, and the increase was larger than observed in Western Europe (+2.6%, 95% CI: +2.3% to +2.9% per year) (Table 3). Again, findings show considerable variation across countries (Fig. 4), ranging from 123.6 pmp in Ukraine to 1080.0 pmp in Greece in 2010, and from 244.1 pmp in Ukraine to 1413.3 pmp in Greece in 2019.

**Table 3:** Unadjusted overall KRT prevalence (on 31 December) pmp by country and year.

Country/year	2010	2011	2012	2013	2014	2015	2016	2017	2018	2019	2020	2021	AAPC 2010–2019	AAPC 2010–2021
Albania		284.0	325.1	340.5	374.4	425.8	507.0	546.8	563.8	602.0	590.6	603.5	**+10.3 (+8.9; +11.8)**	**+8.1 (+6.4; +9.9)**
Belarus^a^						344.5		452.5	432.3	417.1	413.8	439.0	+5.1 (−9.5; +22.2)	+2.8 (−2.2; +8.0)
Bosnia and Herzegovina	738.6	705.0	718.1	746.9	758.9	751.3	758.7	748.8	764.3	763.2	721.3	679.7	**+0.7 (+ 0.1; +1.3)**	−0.6 (−1.6; +0.5)
Bulgaria				540.9	577.5	592.8	610.1	627.1	637.7				**+3.3 (+2.2; +4.2)**	**+3.3 (+2.2; +4.2)**
Croatia														
Cyprus														
Czech Republic	961.0	963.0	996.2	1033.5	1069.4	1111.8	1097.8	1176.0	1221.4	1101.4	1076.2	1126.8	**+2.1 (+1.4; +2.9)**	**+1.2 (+0.4; +1.9)**
Estonia	530.6	532.8	554.0	572.1	634.4	660.6	698.4	720.4	743.6	783.8	789.0	826.5	**+4.8 (+4.2; +5.4)**	**+4.1 (+2.8; +5.4)**
Greece	1080.0	1103.1	1135.7	1172.1	1202.8	1234.6	1284.4	1318.8	1366.4	1413.3	1417.8	1445.4	**+3.1 (+2.9; +3.2)**	**+2.7 (+2.4; +2.9)**
Lithuania				719.0	729.2	754.2	759.3	796.3	833.7	867.5	865.0	855.6	**+3.2 (+2.6; +3.8)**	**+2.7 (+2.0; +3.3)**
Latvia	440.6	517.8	538.2	600.3	626.6	639.6	665.4	683.8	777.6	801.7	605.5	600.4	**+6.5 (+4.5;+ 8.1)**	**+1.6 (+0.2; +2.9)**
North Macedonia	731.0	758.2			763.1	790.3	823.4	870.9	868.4	892.5	851.8	789.9	+2.2 (+1.7;+2.8)	+0.6 (−0.6; +1.8)
Poland	727.0	706.7	747.5	822.4	856.0	800.7	811.8	787.9	885.0	888.0	844.0	848.8	**+2.2 (+0.9; +3.4)**	**+1.6 (+0.3; +3.0)**
Romania^b^	563.7	624.1	765.9	816.9	894.0	967.4	1048.2	1142.5	1140.3	1164.2	1206.1	1184.5	**+8.7 (+6.6; +10.9)**	**+7.3 (+6.3; +8.2)**
Russia	185.5	195.7	213.9	241.4		303.0	309.6	333.3	411.4	411.0	427.0		**+9.8 (+8.7; +10.9)**	**+9.4 (+8.4; +10.4)**
Serbia								735.1	849.8	909.2	919.0	896.4		+4.9 (−1.0; +10.9)
Slovakia^c^	572.7	574.9	843.3	857.2	874.6	888.9	921.6	968.5	976.5	1085.3	1165.1	777.9	**+3.4 (+2.7; +4.1)**	**+4.4 (+3.9; +4.7)**
Türkiye			815.6	870.2	917.9	935.5	933.1	956.7	988.4	1007.4	996.8	993.5	**+2.9 (+1.9; +3.9)**	**+2.4 (+1.3; +3.6)**
Ukraine^a,d^	123.6	130.8	146.7	159.0	157.1	178.0	188.3	209.9	228.8	244.1	267.9	303.6	**+7.8 (+7.1; +8.6)**	**+8.2 (+7.1; +8.6)**
Total Eastern Europe	426.2	438.3	465.0	497.5	519.0	552.9	564.7	596.3	645.2	651.2	653.0	669.1	**+5.1 (+4.5; +5.7)**	**+4.2 (+3.8; +4.6)**
Total Western Europe^e^	984.4	1019.8	1041.9	1048.6	1075.9	1123.4	1150.3	1177.4	1198.2	1253.9	1212.0	1221.6	**+2.6 (+2.3; +2.9)**	**+1.9 (+1.7; +2.2)**

If cells are left empty, data are unavailable.

^a^Patients younger than 18 years of age are not reported for Belarus (2019–2021) and Ukraine (2016–2021).

^b^The overall prevalence of KRT is underestimated by ∼3% due to an estimated 30% underreporting of patients living on a functioning graft;

^c^Data for 2010, 2011, and 2021 include dialysis patients only.

^d^Data for 2010 do not include the regions Zakarpattya, Zaporizzha, and Kiev city, and data for 2011 do not include Kiev city.

^e^Including: Austria, Belgium (Dutch-speaking and French-speaking), Denmark, Finland, France, Iceland, Norway, Portugal, Spain, Sweden, Switzerland, the Netherlands, and the UK (England, Northern Ireland, Scotland, and Wales).

Bold AAPC values depict the statistically significant trends.

Between 2010 and 2019, KRT prevalence increased in every single country, except for Belarus in which the prevalence remained stable (Fig. 4; Table 3).

When follow-up was extended to 2021 KRT prevalence continued to increase in most countries, albeit to a lesser extent. However, in addition to Belarus, KRT prevalence also stabilized in Bosnia and Herzegovina, Czech Republic, North Macedonia, and Serbia (Table 3).

#### Treatment modality

The distribution of treatment modalities of prevalent KRT patients also showed large country differences. In most countries, most patients were treated with dialysis; however, approximately half the prevalent KRT patients in Belarus, Czech Republic, Estonia, Latvia, and Lithuania were living with a functioning kidney transplant (Fig. 4).

The increase in KRT prevalence seemed to be mainly driven by the kidney transplantation prevalence, which increased between 2010 and 2019 in all countries except for Belarus (Table 4). Significant increasing trends in the prevalence of dialysis were observed for 13 of the 19 countries: Albania, Bulgaria, Czech Republic, Estonia, Greece, Lithuania, Latvia, North Macedonia, Romania, Russia, Slovakia, Türkiye, and Ukraine (Table 5). Whereas most of this increase was due to increases in the prevalence of hemodialysis, in Lithuania, Latvia, Russia, and Ukraine the prevalence of peritoneal dialysis (PD) also increased. By contrast, in Bulgaria, Czech Republic, North Macedonia, Poland, and Slovakia PD prevalence decreased over time (Supplementary Table 2).

**Table 4:** Unadjusted prevalence of kidney transplantation (on 31 December) pmp by country and year.

Country/year	2010	2011	2012	2013	2014	2015	2016	2017	2018	2019	2020	2021	AAPC 2010–2019	AAPC 2010–2021
Albania		74.0	72.6	65.4	73.0	80.7	87.8	96.9	105.9	117.9	107.9	110.3	**+5.7 (+5.3; +6.2)**	**+5.9 (+4.0; +7.8)**
Belarus^a^						131.1		187.8	196.6	192.4	203.1	240.3	+10.6 (−4.8; +28.6)	**+8.9 (+3.7; +14.4)**
Bosnia and Herzegovina	47.3	46.5	51.6	56.2	63.0	78.2	88.4	96.9	102.8	107.9	109.3	110.4	**+11.3 (+9.6; +13.1)**	**+8.3 (+6.4; +10.2)**
Bulgaria				69.3	83.7	79.3	82.5	93.3	90.1				**+4.9 (+0.8;+9.2)**	N/A
Croatia														
Cyprus														
Czech Republic	361.0	371.0	394.8	420.9	442.8	464.0	441.1	493.7	521.3	500.0	522.4	522.8	**+4.0 (+2.8; +5.2)**	**+3.3 (+2.6; +4.1)**
Estonia	297.0	302.2	328.0	346.0	366.7	377.8	396.0	411.4	441.8	464.2	496.4	521.4	**+5.1 (+4.8; +5.5)**	**+5.3 (+5.0; +5.5)**
Greece	213.0	220.5	231.3	239.0	243.5	242.4	237.9	242.1	243.6	248.9	255.5	266.6	**+1.6 (+0.9; +2.3)**	**+2.0 (+1.4; +2.6)**
Lithuania				227.8	249.7	282.1	288.1	307.2	317.2	340.7	362.2	385.5	**+6.8 (+5.5; +8.2)**	**+6.7 (+5.8; +7.5)**
Latvia	205.5	276.1	290.0	324.8	338.4	344.3	356.4	372.3	446.7	451.0	318.1	319.9	**+8.4 (+5.2; +10.8)**	+1.9 (0.0; +3.9)
North Macedonia	69.2	70.7			115.2	105.3	102.4	107.8	123.1	109.8	111.7	115.5	**+6.1 (+2.7; +9.7)**	**+5.1 (+2.8; +7.5)**
Poland	222.0	240.6	254.2	281.1	294.9	276.6	286.7	278.4	305.0	305.0	332.0	342.0	**+3.7 (+2.0; +5.4)**	**+4.0 (+3.6; +4.5)**
Romania^b^	39.8	47.0	57.9	64.1	74.2	82.5	91.4	98.4	96.0	98.0	104.1	100.6	**+10.0 (+8.0; +12.0)**	**+9.1 (+7.0; +11.2)**
Russia	41.2	41.5	43.9	47.8		56.4	59.3	64.4	73.0	69.9	74.0		**+7.1 (+6.1; +8.2)**	**+6.8 (+5.9; +7.8)**
Serbia								121.1	137.6	137.3	131.6	125.2		+0.2 (−7.8; +8.8)
Slovakia			248.2	248.2	270.8	273.9	301.6	314.0	317.2	404.6	447.0		**+6.4 (+4.1; +8.7)**	**+8.2 (+5.5; +10.0)**
Türkiye				123.9	143.1	162.6	178.9	189.7	210.0	230.3	232.1	244.0	**+10.7 (+9.0; +12.5)**	**+9.0 (+7.0; +11.1)**
Ukraine^a,c^	17.0	17.2	18.5	20.1	19.7	25.0	27.4	29.7	32.4	34.8	36.7	35.4	**+9.1 (+7.6; +10.7)**	**+8.4 (+7.1; +9.7)**

If cells are left empty data are unavailable.

^a^Patients younger than 18 years of age are not reported for Belarus (2019–2021) and for Ukraine (2016–2021);

^b^Underestimated by ∼3% due to an estimated 30% underreporting of patients living on a functioning graft;

^c^Data do not include the regions Zakarpattya, Zaporizzha and Kiev city (2010) and Kiev city (2011).

Bold AAPC values depict the statistically significant trends.

**Table 5:** Unadjusted prevalence of dialysis (on 31 December) pmp by country and year.

Country/year	2010	2011	2012	2013	2014	2015	2016	2017	2018	2019	2020	2021	AAPC 2010–2019	AAPC 2010–2021
Albania		210	252.5	275.1	296.2	338.1	407.6	442.2	445.9	475.7	475.6	493.2	**+10.3 (+7.0; +13.7)**	**+8.7 (+7.0; +10.3)**
Belarus^a^						213.5		264.6	235.6	224.7	210.7	198.7	+1.3 (−13.6; +18.7)	−2.1 (−7.6; +3.7)
Bosnia and Herzegovina	688.7	658.2	666.2	690.7	695.9	673.2	670.3	651.8	661.6	655.4	612.0	569.2	−0.4 (−0.9; +0.2)	**−0.6 (−1.1; −0.1)**
Bulgaria				471.7	481.2	513.5	527.6	533.8	540.7				**+3.0 (+1.8; +4.2)**	N/A
Croatia	659.4	666.0	646.6	620.4	603.5	597.3	587.7	738.1	624.3	566.2	589.4	647.5	−0.8 (−2.7; +1.1)	−0.6 (−1.9; +0.8)
Cyprus														
Czech Republic	600.0	582.0	601.4	612.6	626.6	647.8	656.7	682.2	700.2	601.4	553.8	604.0	**+0.8 (+0.2; +1.8)**	−0.1 (−1.0; +0.8)
Estonia	233.6	230.6	226.0	226.1	267.8	282.9	302.5	309.0	301.8	319.6	292.6	305.0	**+4.5 (+3.0; +6.0)**	+2.5 (−0.3; +5.3)
Greece	867.0	882.5	904.3	933.1	959.3	992.1	1046.5	1076.7	1122.7	1164.4	1162.4	1178.8	**+3.4 (+3.1; +3.7)**	**+2.9 (+2.4; +3.3)**
Lithuania				491.2	479.5	472.1	471.3	489.1	516.5	526.8	502.8	475.1	**+1.4 (+0.4; +2.4)**	+0.5 (−0.7; +1.8)
Latvia	235.0	241.7	248.2	275.5	288.1	295.3	309.0	311.5	330.9	350.8	287.4	280.5	**+4.5 (+3.8; +5.3)**	**+1.3 (+0.3; +2.2)**
North Macedonia	661.8	687.5			647.8	684.9	721.1	763.1	745.3	782.7	740.1	674.3	**+1.7 (+0.4; +3.0)**	0.0 (−1.9; +1.9)
Poland	505.0	466.1	493.3	541.3	561.2	524.1	525.1	509.4	561.6	555.9	512.3	508.8	+1.3 (−0.0; +2.6)	+0.5 (−0.9; +2.1)
Romania	523.9	576.9	707.8	752.5	819.2	884.2	956.0	1043.4	1043.2	1065.5	1104.4	1083.2	**+8.5 (+6.4; +10.7)**	**+7.1 (+6.2; +8.0)**
Russia	144.3	154.2	170.0	193.6		246.5	250.2	269.0	338.5	341.0	353.0		**+10.5 (+9.2; +11.8)**	**+10.1 (+8.9; +11.3)**
Serbia								602.0	701.0	762.7	780.6	766.9	+0.7 (−1.3; +2.7)	**+6.1 (+0.4; +11.6)**
Slovakia	572.7	574.9	595.0	609.0	603.7	615.0	620.1	654.6	659.2	678.5	718.1	777.9	**+1.9 (+1.5; +2.2)**	**+7.6 (+2.7; +12.7)**
Türkiye				746.3	747.4	772.9	754.2	767.0	778.4	777.3	764.8	749.5	**+0.7 (+0.2; +1.2)**	0.0 (−0.9; +1.0)
Ukraine^a,b^	106.5	113.7	128.3	138.9	137.5	152.9	160.9	180.3	196.4	209.4	231.2	268.3	**+7.6 (+6.9; +8.4)**	**+8.2 (+7.5; +9.0)**

If cells are left empty, data are unavailable

^a^Patients younger than 18 years of age are not reported for Belarus (2019–2021) and for Ukraine (2016–2021);

^b^Data do not include the regions Zakarpattya, Zaporizzha, and Kiev city (2010 and 2011).

Bold AAPC values depict the statistically significant trends.

#### Age

**Figure 5:**
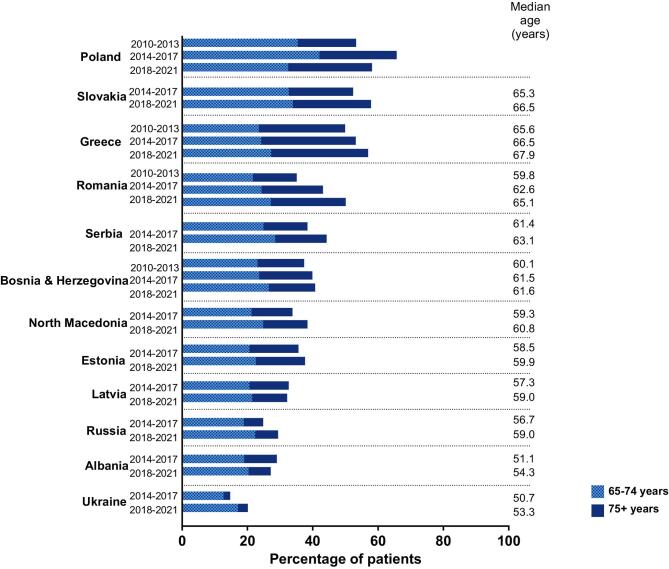
Proportion of prevalent KRT patients (31 December) aged 65 years or older and their median ages by country and period. Patients younger than 18 years of age are not reported for Ukraine in 2016–2021. Data include dialysis patients only for Slovakia (2010–2021). The overall KRT prevalence in Romania is underestimated by ∼3% due to an estimated 30% underreporting of patients living on a functioning graft.

Median age of prevalent KRT patients varied from 50.7 to 53.3 years over the time periods in patients from Ukraine to 65.6 to 67.9 years in patients from Greece, but increased over time in all countries, as did the percentage of prevalent KRT patients aged 65 years or older (Fig. 5).

#### Sex and primary renal disease

In all countries, except for Bulgaria (similar prevalence for both sexes), KRT prevalence was consistently higher among males than among females, without major sex differences in trends over time (Table 6; Supplementary Fig. 2).

**Table 6:** Trends (AAPC) from 2010 to 2019 in unadjusted KRT prevalence (on 31 December) pmp by country stratified by sex and PRD.

	Sex		PRD				
Country	Male	Female	GN	DM	HT	Unknown	Other
Albania	**+10.1 (+8.6; +11.6)**	**+10.5 (+8.1; +12.8)**	**+5.8 (+1.4; +10.4)**	**+29.6 (+23.5; +36.0)**	**+29.8 (+23.2; +36.7)**	**−3.4 (−6.2; −0.5)**	**+10.5 (+8.8; +12.3)**
Belarus^a^	+6.6 (−5.5; +20.1)	+3.5 (−14.6; +25.4)	+3.6 (−11.2; +21.0)	+3.5 (−12.9; +22.9)	**+21.2 (+3.3; +42.2)**	−3.3 (−55.6; +110.5)	+4.6 (−13.2; +26.0)
Bosnia and Herzegovina	**+1.4 (+0.8; +2.0)**	−0.4 (−0.8;0.0)	−0.1 (−0.5; +0.4)	**+4.2 (+2.9; +5.6)**	**+7.8 (+6.2; +9.4)**	**+4.2 (+0.8; +7.8)**	**−2.1 (−3.0; −1.2)**
Bulgaria	**+3.6 (+3.0; +4.3)**	**+2.8 (+0.3; +5.2)**					
Croatia							
Cyprus							
Czech Republic							
Estonia^b^	**+4.6 (+3.2; +6.0)**	**+4.3 (+2.6; +6.0)**	**+4.1 (+3.4; +4.8)**	**+3.9 (+3.2; +4.6)**	+1.5 (−6.9; +10.7)	N/A	**+7.1 (+1.9; +12.6)**
Greece	**+3.6 (+3.4; +3.7)**	**+2.3 (+2.0; +2.6)**	**+1.3 (+1.1; +1.4)**	**+3.3 (+2.9; +3.7)**	**+2.8 (+2.0; +3.5)**	**+4.1 (+3.8; +4.4)**	+1.1 (−2.0; +4.4)
Lithuania							
Latvia	**+6.5 (+5.3; +7.7)**	**+5.7 (+4.5; +6.9)**	**+4.8 (+2.8; +6.8)**	**+6.7 (+0.4; +13.4)**	**+11.0 (+5.3; +17.0)**	**+6.2 (+3.2; +9.3)**	**+5.9 (+4.5; +7.3)**
North Macedonia	**+2.5 (+1.5; +3.5)**	**+1.9 (+1.4; +2.5)**	−0.3 (−2.4; +1.9)	**+6.4 (+5.4; +7.5)**	**+6.4 (+3.7; +9.1)**	−1.6 (−4.7; +1.6)	+0.8 (−0.2; +1.9)
Poland	+4.6 (−3.0; +12.8)	+1.9 (−0.7; +4.6)	+0.7 (−2.3; +3.9)	**+5.1 (+2.5; +7.7)**	+2.4 (−1.6; +6.3)	+1.5 (−0.4; +3.3)	**+1.2 (+0.4; +2.1)**
Romania^c^	**+8.9 (+6.8; +11.1)**	**+7.8 (+5.6; +10.0)**	**+4.8 (+3.1; +6.5)**	**+9.7 (+5.0; +14.6)**	**+9.5 (+7.3; +11.7)**	**+12.0 (+9.0; +15.2)**	+4.6 (−0.6; +10.0)
Russia	**+10.3 (+8.1: +12.5)**	**+9.4 (+7.7; +11.1)**	**+7.9 (+4.4; +11.4)**	**+16.1 (+15.1; +17.1)**	**+19.7 (+10.1; +30.1)**	**−10.8 (−18.1; −2.8)**	**+10.9 (+9.3; +12.6)**
Serbia							
Slovakia^d^	**+2.1 (+1.7; +2.6)**	**+1.4 (+0.8; +2.0)**	+1.5 (−0.4; +3.4)	+1.0 (−0.6; +2.7)	+1.2 (−3.6; +6.3)	+4.5 (−0.3; +9.4)	**+2.4 (+0.9; +4.0)**
Türkiye							
Ukraine^a,e^	**+8.0 (+7.2; +8.7)**	**+7.8 (+6.9; +8.7)**	**+4.9 (+3.8; +6.1)**	**+10.9 (+9.6; +12.2)**	**+14.1 (+10.4; +17.9)**	**+21.7 (+11.2; +33.2)**	**+9.0 (+8.2; +9.7)**

If cells are left empty, data are unavailable.

^a^Patients younger than 18 years of age are not reported for Belarus (2019) and for Ukraine (2016–2019).

^b^For 2012 renal vascular disease was not reported separately, but included into hypertension.

^c^The overall prevalence of KRT is underestimated by ∼3% due to an estimated 30% underreporting of patients living on a functioning graft.

^d^Data include dialysis patients only.

^e^Data do not include the regions Zakarpattya, Zaporizzha, and Kiev city (2010 and 2011).

Bold AAPC values depict the statistically significant trends.

Abbreviation: GN, glomerulonephritis.

**Figure 6:**
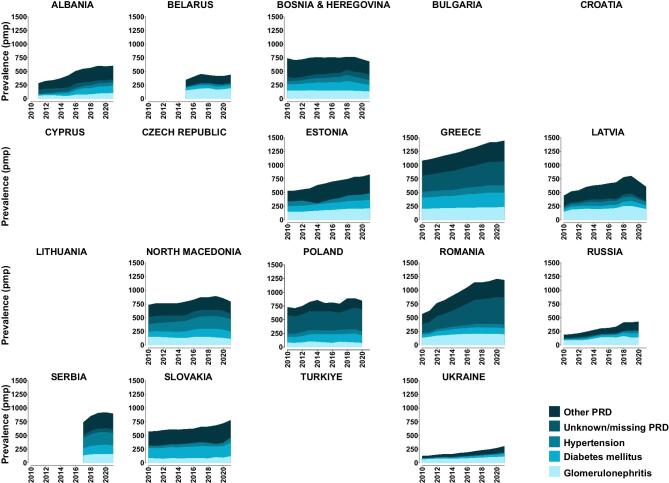
Unadjusted KRT prevalence (31 December) (pmp) by country and year; stratified by PRD. Data are unavailable for Bulgaria, Croatia, Cyprus, Czech Republic, Lithuania, and Türkiye. Patients younger than 18 years of age are not reported for Belarus (2019–2021) and for Ukraine (2016–2021). The overall KRT prevalence in Romania is underestimated by ∼3% due to an estimated 30% underreporting of patients living on a functioning graft. Data for Slovakia include dialysis patients only. Data for Ukraine do not include the regions Zakarpattya, Zaporizzha, and Kiev city (2010 and 2011).

The PRD distribution of prevalent patients significantly varied across the region. For example, in Belarus, Russia, and Ukraine, glomerulonephritis was present in almost half of patients, whereas this proportion was much lower for other countries. However, the proportion of unknown/missing PRDs was also highly variable across countries (Fig. 6).

For most countries the KRT prevalence (pmp) of every PRD category (including unknown or missing causes) increased between 2010 and 2019, but the largest increases were observed for prevalent KRT patients with DM and HT (Fig. 6, Table 6).

## DISCUSSION

Between 2010 and 2019, the overall KRT incidence increased at 1.5% per year in Central and Eastern Europe, while KRT prevalence increased at 5% per year. But there were large variations between individual countries. The median age of both the incident and prevalent KRT population increased over the study period, indicating that an increasing number of older people were receiving KRT over time. The largest increases were observed for patients with diabetes or hypertension as the cause of kidney failure. The COVID-19 pandemic did not seem to have a substantial impact on KRT incidence and prevalence in the region, as most trends remained similar, although they were less pronounced.

### Incidence of KRT

Overall KRT incidence in Central and Eastern Europe increased at 1.5% per year between 2010 and 2019, which was similar to the 1.4% increase found in Western Europe. Remarkably, in 2019, KRT incidence varied by 7-fold in the region, while it varied only 3-fold in Western Europe [18]. Similarly, macro-economic factors, which are strongly associated with the access to KRT for patients with kidney failure [5, 9, 10, 20], showed much greater variation in Eastern and Central European countries. Nevertheless, the percentage of pre-emptive kidney transplantations in incident KRT patients appeared to be low (∼5%) both in Central/Eastern and Western Europe [18].

Over time KRT incidence increased or stabilized in all countries except in Bosnia and Herzegovina in which KRT incidence significantly decreased over time. While these decreases are believed to be multifactorial, exact reasons remain unknown. However, several factors explaining country differences in KRT incidence might be at play. CKD risk factors, prevalence, and progression rate, as well as access to KRT, clearly differ by country and are affected by both general population health and by the organization of healthcare delivery [4, 5, 19, 20]. Paradoxically, in Central and Eastern Europe CKD prevalence was inversely associated with KRT incidence [20] and prevalence [20, 21], possibly due to poorer population health and inadequate healthcare capacity [20]. Indeed, life expectancy (a marker of general population health [22]) was related to KRT differences across countries [21] and affordable and equitable general healthcare will result in higher life expectancy, higher survival rates, and hence more and older patients reaching kidney failure and requiring KRT [21]. Other organizational differences in kidney care delivery contributing to country differences in KRT incidence may include the share of private and public dialysis centers, patient and nephrologist preferences, use of cardio- and renal protective drugs, and differences in clinical practice patterns, such as the timing of dialysis initiation and the availability of comprehensive conservative management (CCM) [4, 5, 7, 19, 23–26]. CCM is offered as alternative to KRT in several Central and Eastern European countries [27, 28], but its use was related to cultural beliefs, patient mentality, and income-level [28, 29] and lower in Eastern compared to Western Europe [28]. Notably, the ERA Registry collects data on patients with kidney failure who are treated with KRT and information on CKD patients with kidney failure who remain untreated or who receive CCM is lacking. Furthermore, developments in the general population, including migration and the uptake of refugees, might have contributed to country variations and trends in KRT incidence rates.

Although increasing in most Central and Eastern European countries, median age at KRT initiation showed considerable variation across individual countries. For example, in Ukraine (lowest KRT incidence) median age at KRT start was 20 years lower (52–55 years) compared to Greece (highest KRT incidence), a larger variation than observed for Western European countries, where median age differed up to 15 years [18]. This remarkable difference can again, at least partly, be explained by country macro-economics. When limited resources are available mainly younger and healthier patients will be accepted for KRT [4]. In addition, the most pronounced variation across countries was observed in the proportion of elderly patients (75 years or older) commencing KRT. When an increasing number of older patients are treated, KRT incidence will be higher [30]. Interestingly, there was a higher male predominance in countries with higher KRT incidence and sex differences became larger with increasing age as shown in a previous ERA Registry analysis [18]. In turn, the age differences across countries might also be related to differences in the PRD distribution of incident KRT patients. In countries with a younger KRT population the proportion of patients with glomerulonephritis was substantially higher (for example Russia and Ukraine) than among countries treating older patients (i.e. Greece and Cyprus). It should be noted that most diagnoses are probably not biopsy-proven with discrepancies between actual and reported diagnoses. In addition, high proportions of unknown or missing causes of kidney failure were more common in Central and Eastern Europe than in Western Europe [18], possibly partly due to late referral [4].

### Prevalence of KRT

Overall in Central and Eastern Europe, KRT prevalence increased at 5% per year from 2010 to 2019, which was twice as high as the increase observed in Western Europe (APC: +2.5% between 2011 and 2017, and +2.6% from 2010 to 2019) [10]. In 2019, KRT prevalence in Central and Eastern Europe was almost half of that in Western Europe, and the treatment modality distribution clearly differed [18]. While most prevalent patients in Central and Eastern Europe were receiving HD (66%) and 29% of patients were living with a functioning transplant, in Western Europe nearly half of prevalent patients were receiving HD (48%) and the other half was living with a functioning graft (47%) [18]. Also within Central and Eastern Europe the treatment modality distribution among prevalent patients differed. Treatment modality choice can be influenced by several factors, including patient- and nephrologist-related factors. Two surveys regarding treatment modality choice among European kidney failure patients [25] and nephrologists [24] showed limited availability of treatments and information provision on treatment options other than center HD in countries with lower gross domestic product [24, 25]. Similarly, a country’s economic situation seemed to be a strong determinant for the proportional use of home-based dialysis therapies and kidney transplantation [29]. Despite advantages for both patients and healthcare budgets, compared to Western Europe, PD use is still low in many, but not all, Central and Eastern European countries [14, 25, 29]. Lack of (patient) education and well-trained staff, but also financial incentives by the HD industry seem to be major barriers to provide PD and home hemodialysis [31–33]. The recent appearance of epidemiological data and initiatives to increase the uptake of home-dialysis therapies might result in a higher future use within the region [31, 32]. In several countries, i.e. Lithuania, Czech Republic, Belarus, Latvia, and Estonia 40%–60% of all prevalent KRT patients in 2019 were living with a kidney transplant. A recent ERA Registry analysis showed that the annual kidney transplant rate between 2010 and 2018 increased in most European countries, both in Eastern and Western Europe [34]. However, an east–west gradient was observed for the number of performed kidney transplantations from deceased donors, with lower rates in Eastern Europe. Nevertheless, in several Eastern European countries (including Belarus, Croatia, and Türkiye) initiatives to increase kidney transplantation rates were successfully implemented by their Ministry of Health [34–37]. These initiatives, including the use of expanded donor criteria transplants, education of professionals, raising public awareness, and financial incentives for living donors [38], show possibilities to further increase kidney transplantation within this region.

Similar to other regions around the globe [39], an increasing KRT prevalence was observed in all, but one (Belarus) countries in Central and Eastern Europe, and this increase basically means that the number of deaths on KRT is lower than the number of patients commencing KRT. Furthermore, the increasing trend in KRT prevalence was mainly driven by the increase in the share of patients living with functioning grafts and to a lesser extent by the number of prevalent dialysis patients. Nevertheless, developments in the general population, such as migration, might have contributed to country variations and trends in KRT prevalence. Because of population aging together with lower (non-CKD related) mortality, the KRT prevalence in Central and Eastern Europe is expected to further increase over the next decades [21, 40].

### Effect of COVID-19

The overall effect of the COVID-19 pandemic on KRT incidence and prevalence in the region seemed to be limited. For most countries, trends in KRT incidence and prevalence until the end of 2021, including the 2 years of the COVID-19 pandemic, remained but were generally less pronounced when compared to the pre-COVID era. In a recent ERA Registry study, mainly including Western European countries, a significant decrease in the KRT incidence (6.2% lower in 2020 vs. 2017–2019) and only a modest increase in the KRT prevalence (+0.2% in 2020 vs. 2019) was reported during the pandemic [41]. When comparing the same periods in our study, we found a similar decrease in incidence in 2020 vs. 2017–2019 (−5.7%), whereas the prevalence was also only marginally higher in 2020 than in 2019 (+0.1%). Since trends in KRT incidence and prevalence from 2010 to 2021 were not very different from trends from 2010 to 2019, there seemed to be some catch-up effect during 2021.

### Strengths and limitations

This study is the first to provide a complete overview on trends in the epidemiology of KRT in Central and Eastern Europe in the past decade that can assist policy makers and the medical community in healthcare planning. However, some limitations of our work need to be acknowledged. Data on KRT patients from Central and Eastern Europe were mainly collected by the ERA Registry on aggregated country level rather than on individual patient level. Cases that were reported after this date (late reporting) were not accounted for in aggregated data collections. Only some countries reported data by patient subgroups (age, sex, PRD, and treatment modality) and data on patient mortality was unavailable. We cannot exclude the possibility that changes in data collection processes within the participating countries may have occurred, potentially influencing the observed trends in KRT. However, at the ERA Registry extensive annual quality checks, partly aimed at detecting unexpected changes, have been performed after receipt of each data set, ensuring a high level of data consistency over time.

## CONCLUSION

This is the first overview of KRT incidence and prevalence trends over the past decade for Central and Eastern Europe. Both KRT incidence (+1.5% per year) and prevalence (+5% per year) significantly increased in the region from 2010 to 2019, with no major impact of the COVID-19 pandemic in 2020 and 2021. The median age of the KRT population also increased. Our study highlights important variation in KRT incidence and prevalence across the region, which was much larger than differences between countries in Western Europe. Although increasing in most individual countries, kidney transplantation numbers are still behind numbers achieved in Western Europe and further development of kidney transplantation programs should be given high priority. This study is a first important step to define country-specific priorities for the optimization of kidney care and to create further public awareness for kidney diseases in the Central and Eastern European region.

## Supplementary Material

gfaf268_Supplemental_File

## Data Availability

The data underlying this article cannot be shared with any third party because the national and regional registries that provided data to the ERA Registry remain the owners of the data.
