# Supplementary material for: Incidence and prevalence of kidney replacement therapy in Central and Eastern Europe—trends from the ERA Registry
Source: Nephrol Dial Transplant. 2026 Jan 7;41(7):1322–39. doi: 10.1093/ndt/gfaf268 (PMC13403274; doi:10.1093/ndt/gfaf268)
Supplement: gfaf268_Supplemental_File [file gfaf268_Supplemental_File.docx]

**Supplementary material**

**Table S1. Percentage coverage of the general population by country and year**

| Country/year | **2010** | **2011** | **2012** | **2013** | **2014** | **2015** | **2016** | **2017** | **2018** | **2019** | **2020** | **2021** |
| --- | --- | --- | --- | --- | --- | --- | --- | --- | --- | --- | --- | --- |
| Albania |  | 100 | 100 | 100 | 99.0 | 99.0 | 99.0 | 99.0 | 99.0 | 99.0 | 99.0 | 99.0 |
| Belarus |  |  |  |  |  | 98.0 |  | 100 | 100 | 100 | 100 | 100 |
| Bosnia & Herzegovina | 100 | 100 | 100 | 100 | 100 | 100 | 100 | 100 | 100 | 100 | 100 | 100 |
| Bulgaria |  |  |  | 97.0 | 98.0 | 100 | 100 | 100 | 100 |  |  |  |
| Croatia | 100 | 100 | 100 | 80.0 | 95.0 | 80.0 | 90.0 | 90.0 | 90.0 | 85.0 | 85.0 | 85.0 |
| Cyprus |  |  |  | 100 | 100 | 100 | 100 | 100 | 100 | 100 | 100 | 100 |
| Czech Republic | 99.0 | 99.0 | 99.0 | 98.0 | 97.0 | 98.0 | 97.0 | 93.5 | 93.8 | 98.0 | 98.0 | 98.0 |
| Estonia | 100 | 100 | 100 | 100 | 100 | 100 | 100 | 100 | 100 | 100 | 100 | 100 |
| Greece | 100 | 100 | 100 | 100 | 100 | 100 | 100 | 100 | 100 | 100 | 100 | 100 |
| Latvia | 100 | 85.5 | 85.0 | 80.0 | 80.0 | 80.0 | 80.0 | 80.0 | 68.0 | 68.0 | 87.0 | 90.0 |
| Lithuania |  |  |  | 100 | 100 | 100 | 100 | 100 | 100 | 100 | 100 | 100 |
| North Macedonia | 100 | 100 |  |  | 100 | 100 | 100 | 100 | 100 | 100 | 100 | 100 |
| Poland | 95.0 | 100 | 98.0 | 95.0 | 95.0 | 99.0 | 100 | 100 | 100 | 100 | 100 | 100 |
| Romania | 100 | 100 | 96.0 | 99.0 | 99.0 | 99.0 | 99.0 | 97.0 | 97.9 | 98.6 | 95.0 | 100 |
| Russia | 100 | 100 | 99.0 | 100 |  | 99.0 | 98.0 | 97.0 | 91.0 | 98.0 | 98.0 |  |
| Serbia |  |  |  |  |  |  |  | 97.0 | 90.0 | 90.0 | 90.0 | 95.0 |
| Slovakia | 97.0 | 100 | 100 | 100 | 100 | 100 | 100 | 100 | 100 | 82.0 | 79.2 | 81.8 |
| Türkiye |  |  | 100 | 100 | 100 | 100 | 100 | 100 | 100 | 100 | 100 | 100 |
| Ukraine | 99.0 | 94.3 | 100 | 100 | 100 | 100 | 100 | 100 | 100 | 100 | 100 | 64.3 |

If cells are left empty data are unavailable.

**Table S2. Unadjusted prevalence of hemodialysis (on December 31) per million population (pmp) by country and year**

| **Country/year** | **2010** | **2011** | **2012** | **2013** | **2014** | **2015** | **2016** | **2017** | **2018** | **2019** | **AAPC 2010-2019** |
| --- | --- | --- | --- | --- | --- | --- | --- | --- | --- | --- | --- |
| Albania |  | 194.6 | 234.6 | 257.1 | 279.4 | 320.2 | 391.2 | 425.3 | 431.1 | 461.6 | **+11.0 (+9.2;+13.2)** |
| Belarus^1^ |  |  |  |  |  | 183.6 | 161.8 | 230.6 | 204.6 | 201.1 | +4.3 (-5.5; +14.6) |
| Bosnia & Herzegovina | 657.1 | 632.0 | 642.8 | 666.5 | 672.8 | 647.4 | 644.8 | 627.6 | 634.4 | 627.6 | +0.2 (-1.2; +1.6) |
| Bulgaria |  |  |  | 449.4 | 458.9 | 491.3 | 506.6 | 514.5 | 522.1 |  | **+3.3 (+1.4; +5.9)** |
| Croatia |  |  |  |  |  |  |  |  |  |  |  |
| Cyprus |  |  |  |  |  |  |  |  |  |  |  |
| Czech Republic | 552.5 | 541.0 | 554.4 | 562.3 | 584.4 | 602.6 | 614.9 | 642.4 | 664.2 | 571.1 | **+1.2 (+0.5; +2.3)** |
| Estonia | 176.9 | 179.1 | 194.3 | 192.7 | 230.5 | 245.6 | 260.7 | 261.9 | 255.7 | 266.8 | **+5.3 (+4.1; +6.6)** |
| Greece | 800.2 | 818.7 | 844.4 | 871.8 | 896.4 | 926.8 | 979.2 | 1010.0 | 1055.4 | 1100.5 | **+2.7 (+2.1; +3.4)** |
| Lithuania |  |  |  | 475.4 | 462.5 | 454.6 | 454.3 | 470.5 | 497.3 | 506.8 | **+1.3 (+0.6; +2.1)** |
| Latvia | 185.7 | 195.3 | 195.3 | 221.8 | 230.2 | 236.9 | 246.8 | 248.2 | 253.9 | 276.0 | **+4.3 (+3.3; +5.3)** |
| North Macedonia | 648.9 | 674.6 |  |  | 633.0 | 669.1 | 708.7 | 752.2 | 735.9 | 774.5 | **+1.9 (+1.0; +3.0)** |
| Poland | 474.7 | 438.4 | 463.9 | 513.4 | 532.4 | 495.4 | 500.3 | 480.1 | 537.6 | 535.5 | **+1.5 (+0.1; +3.0)** |
| Romania | 452.8 | 506.5 | 628.9 | 676.7 | 745.1 | 812.5 | 886.9 | 973.2 | 972.7 | 1002.8 | **+9.6 (+8.0; +11.2)** |
| Russia | 131.9 | 141.3 | 156.8 | 179.8 |  | 230.9 | 234.0 | 252.0 | 319.1 | 322.0 | **+10.9 (+8.8; +13.1)** |
| Serbia |  |  |  |  |  |  |  | 538.9 | 626.4 | 682.1 |  |
| Slovakia | 553.4 | 557.7 | 577.5 | 592.9 | 588.6 | 601.0 | 606.3 | 641.0 | 644.0 | 664.7 | **+2.0 (+1.5; +2.5)** |
| Türkiye | 671.5 | 709.5 |  | 687.1 | 692.0 | 723.3 | 710.2 | 725.6 | 739.5 | 737.7 | **+0.9 (+0.3; +1.6)** |
| Ukraine^1,2^ | 92.2 | 97.7 | 109.0 | 117.6 | 118.1 | 131.2 | 139.4 | 159.4 | 175.9 | 187.4 | **+8.4 (+7.1; +9.4)** |

If cells are left empty data are unavailable

^1^Patients younger than 18 years of age are not reported for Belarus (2019-2021) and for Ukraine (2016-2021);

^2^Data do not include the regions Zakarpattya, Zaporizzha and Kiev city (2010) and Kiev city (2011).

Bold AAPC values depict the statistically significant trends.

Abbreviations: Pmp, per million population; AAPC, average annual percentage change.

**Table S3. Unadjusted prevalence of peritoneal dialysis (on December 31) per million population (pmp) by country and year**

| **Country/year** | **2010** | **2011** | **2012** | **2013** | **2014** | **2015** | **2016** | **2017** | **2018** | **2019** | **AAPC 2010-2019** |
| --- | --- | --- | --- | --- | --- | --- | --- | --- | --- | --- | --- |
| Albania |  | 15.4 | 17.9 | 18.0 | 16.8 | 17.9 | 16.4 | 16.9 | 14.8 | 14.1 | -1.0 (-3.3; +1.0) |
| Belarus^1^ |  |  |  |  |  | 29.9 | 14.3 | 34.0 | 31.0 | 23.6 | +3.1 (-20.1; +32.0) |
| Bosnia & Herzegovina | 31.6 | 27.0 | 23.4 | 24.2 | 23.1 | 25.8 | 25.5 | 24.4 | 27.2 | 27.8 | -1.1 (-2.5; +1.1) |
| Bulgaria |  |  |  | 22.3 | 22.3 | 22.2 | 21.0 | 19.3 | 18.6 |  | **-3.9 (-6.5; -1.2)** |
| Croatia |  |  |  |  |  |  |  |  |  |  |  |
| Cyprus |  |  |  |  |  |  |  |  |  |  |  |
| Czech Republic | 47.3 | 49.0 | 47.0 | 50.3 | 42.2 | 45.2 | 41.8 | 39.8 | 36.0 | 30.3 | **-5.3 (6.8; -3.6)** |
| Estonia | 56.7 | 51.5 | 31.7 | 33.4 | 37.3 | 37.3 | 41.8 | 47.1 | 46.1 | 52.8 | -1.9 (-3.9; +0.9) |
| Greece | 66.8 | 63.8 | 59.9 | 61.3 | 62.9 | 65.3 | 67.3 | 66.7 | 67.3 | 63.9 | +0.1 (-1.7; +1.8) |
| Lithuania |  |  |  | 15.8 | 17.0 | 17.5 | 17.0 | 18.6 | 19.2 | 20.0 | **+3.7 (+2.6; +4.8)** |
| Latvia | 49.3 | 46.4 | 52.9 | 53.7 | 57.9 | 58.4 | 62.2 | 63.3 | 77.0 | 74.8 | **+5.4 (+3.8; +6.9)** |
| North Macedonia | 12.9 | 12.9 |  |  | 14.8 | 15.8 | 12.4 | 10.9 | 9.4 | 8.2 | **-5.0 (-5.8; -4.1)** |
| Poland | 30.3 | 27.7 | 29.4 | 27.9 | 28.8 | 28.7 | 24.8 | 29.3 | 24.0 | 20.4 | **-3.9 (-5.3; -2.3)** |
| Romania | 71.1 | 70.4 | 78.9 | 75.8 | 74.1 | 71.7 | 69.1 | 70.2 | 70.5 | 62.7 | -0.7 (-1.8; +0.4) |
| Russia | 12.4 | 12.9 | 13.2 | 13.8 |  | 15.6 | 16.2 | 17.0 | 19.4 | 19.0 | **+5.3 (+4.5; +6.0)** |
| Serbia |  |  |  |  |  |  |  | 63.1 | 74.6 | 80.6 |  |
| Slovakia | 19.3 | 17.2 | 17.6 | 16.1 | 15.1 | 14.0 | 13.8 | 13.6 | 15.2 | 13.8 | **-3.3 (-4.7; -2.0)** |
| Türkiye | 71.4 | 63.3 |  | 59.2 | 55.4 | 49.6 | 44.0 | 41.4 | 38.9 | 39.6 | **-6.8 (-8.7; -4.8)** |
| Ukraine^1,2^ | 14.3 | 16.0 | 19.3 | 21.3 | 19.4 | 21.7 | 21.5 | 20.9 | 20.5 | 22.0 | **+4.4 (+3.1; +6.0)** |

If cells are left empty data are unavailable

^1^Patients younger than 18 years of age are not reported for Belarus (2019-2021) and for Ukraine (2016-2021);

^2^Data do not include the regions Zakarpattya, Zaporizzha and Kiev city (2010) and Kiev city (2011).

Bold AAPC values depict the statistically significant trends.

Abbreviations: Pmp, per million population; AAPC, average annual percentage change.

**Figure S1. Unadjusted incidence of KRT at day 1 per million population (pmp) by country and year; stratified by sex.**

Data are unavailable for Czech Republic, Poland and Türkiye. Patients younger than 18 years of age are not reported for Belarus (2019-2021) and Ukraine (2016-2021). Data include dialysis patients only for Bulgaria (2013-2015), Croatia (2020-2021), Lithuania (2013), Russia (2015-2020), and Slovakia (2010-2021). For Romania the incidence of pre-emptive kidney transplantation is underestimated by approximately 30% for 2017-2021. For Ukraine the data do not include the regions Zakarpattya, Zaporizzha and Kiev city (2010) and Kiev city (2011).

Abbreviations: Pmp, per million population; KRT, kidney replacement therapy.

**Figure S2. Unadjusted KRT prevalence (December 31) per million population (pmp) by country and year; stratified by sex.**

Data are unavailable for Croatia, Cyprus, Czech Republic, Lithuania, and Türkiye. Patients younger than 18 years of age are not reported for Belarus (2019-2021) and Ukraine (2016-2021). The overall KRT prevalence in Romania is underestimated by approximately 3% due to an estimated 30% underreporting of patients living on a functioning graft. Data for Slovakia include dialysis patients only. Data for Ukraine do not include the regions Zakarpattya, Zaporizzha and Kiev city (2010) and Kiev city (2011).

Abbreviations: Pmp, per million population; KRT, kidney replacement therapy.
